# Structural comparisons of the nucleoprotein from three negative strand RNA virus families

**DOI:** 10.1186/1743-422X-4-72

**Published:** 2007-07-10

**Authors:** Ming Luo, Todd J Green, Xin Zhang, Jun Tsao, Shihong Qiu

**Affiliations:** 1 Department of Microbiology, University of Alabama at Birmingham, Birmingham, AL, 35294, USA

## Abstract

Structures of the nucleoprotein of three negative strand RNA virus families, borna disease virus, rhabdovirus and influenza A virus, are now available. Structural comparisons showed that the topology of the RNA binding region from the three proteins is very similar. The RNA was shown to fit into a cavity formed by the two distinct domains of the RNA binding region in the rhabdovirus nucleoprotein. Two helices connecting the two domains characterize the center of the cavity. The nucleoproteins contain at least 5 conserved helices in the N-terminal domain and 3 conserved helices in the C-terminal domain. Since all negative strand RNA viruses are required to have the ribonucleoprotein complex as their active genomic templates, it is perceivable that the (5H+3H) structure is a common motif in the nucleoprotein of negative strand RNA viruses.

## Background

Negative strand RNA viruses are different from all other viruses because their RNA genomes are always enwrapped by a virally coded nucleoprotein (N) to form a ribonucleoprotein (RNP) complex. This complex serves as the template for viral RNA synthesis (the plus strand cRNA, the negative strand vRNA or mRNA) and form the structural core when packaged into virions. The RNP is formed concomitant with replication of viral genomic RNAs by the viral RNA-dependent RNA polymerase. The RNP structure is a unique feature of negative strand RNA viruses such that the polymerase complex can only copy the RNA sequence in the RNP, not naked RNA. In addition, virion assembly also requires the RNP structure to be packaged in the virion. These unique functions that are common among negative strand RNA viruses may require conserved structural motifs in the N protein. Such conservation has been observed in capsid proteins of spherical viruses which share a β-barrel motif [1]. The eight strand β-barrel has the general property of encapsidating nucleotides and self-assembly into an icosahedral shell. Structural similarities have also been noticed in the coat proteins of three other icosahedral virus groups that suggested evolutionary lineages in the virus group. dsDNA virus (e.g. adenovirus), tailed dsDNA viruses (e.g. bacteriophages), and dsRNA viruses (e.g. reovirus) share common protein folds in their coat proteins and common architectures in virion assembly [2].

Crystal structures of the N protein from borna disease virus (BDV) [3], two rhabdoviruses, vesicular stomatitis virus (VSV) [4] and rabies virus (RABV) [5], and influenza A virus (FLUAV) [6], have been reported. The structures of the rhabdovirus N proteins were determined with RNA bound in a cavity. The cavity is located between two separated domains that accommodate the RNA with both hydrophobic and charged/polar interactions. Extended N-termini and a loop in the C-terminal domain reach over neighboring molecules to form an extended protein network along the RNA. The BDV and FLUAV N proteins were determined as a tetramer and a trimer, respectively, in the absence of RNA. The collection of N protein structures from three negative strand RNA viruses makes it possible to identify conserved structural motifs in the nucleoprotein from different virus families. We found that the RNA binding region of the N protein contains an N-terminal domain and a C-terminal domain with a similar topology in the N protein structures of all three virus families. In the RNA binding cavity, a central α-helix surrounded by four α-helices in the N-terminal domain continues to a central α-helix surrounded by two α-helices in the C-terminal domain. By superimposing the rhabdovirus N protein structure with that of BDV and FLUAV, this structural motif was also present in the other two structures. This suggests that the (5H+3H) structure may be a common motif in the nucleoprotein of negative strand RNA viruses.

## Hypothesis

### Superposition of β-barrels in the viral capsid proteins

**Figure 1:**
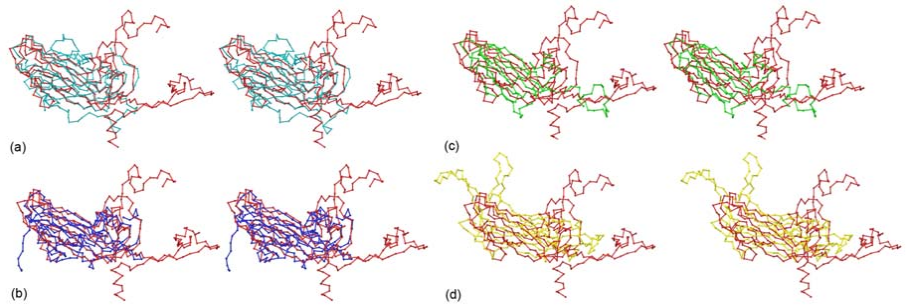
β-barrel comparisons. (a) Stereo Cα drawings for the superposition of the β-barrel fold in HRV16-VP2 (cyan) with that in HRV16-VP1 (red). (b) SBMV (blue) with HRV16-VP1 (red). (c) STMV (green) with HRV16-VP1 (red). (d) HPV (yellow) with HRV16-VP1 (red). In this and the following figures, the Cα tracing was prepared with RIBBONS [14] and protein structural cartoons were prepared with PyMol [15].

The β-barrel structural motif of spherical viruses can serve as benchmarks for recognizing conserved structural folds in other viral proteins. Four spherical viruses were selected, including human rhinovirus serotype 16 (HRV16, PDB accession code 1AYM) viral protein 1 (VP1) and viral protein 2 (VP2) [7], a single stranded RNA virus, the C-subunit of the southern bean mosaic virus (SBMV, coordinates retrieved from the Protein Data Bank (PDB) with accession code 4SBV([8], a plant RNA virus, capsid protein of satellite tobacco mosaic virus (STMV, PDB accession code 1A34) [9], a small plant RNA virus, and the L1 protein of human papillomavirus (HPV, PDB accession code 1DZL) type 16 [10], a double stranded DNA virus. The coordinates of HRV16-VP2 and the last three viral capsid proteins were aligned with that of HRV16-VP1 using FATCAT (Figure 1) and the results are tabulated in Table 1. The structural alignment of the coordinates in each comparison was carried out by the method of Flexible structure AlignmenT by Chaining AFPs (Aligned Fragment Pairs) with Twists (FATCAT) [11,12].

**Table 1:** Structural alignment of the β-barrel fold

RMSD (Å) P-value Aligned residues	HRV16-VP2 (252 residues)	SBMV C-sub (222 residues)	STMV (147 residues)	HPV (233 residues^≠^)
HRV16-VP1 (285 residues)	**3.06** **1.57e-3** **145**	**3.02** **1.63e-3** **149**	**3.50** **1.63e-1** **108**	**3.56** **1.16e-1** **111**

≠In the L1 capsid protein of HPV, three large loops and the C-terminal extension were removed to isolate the β-barrel fold for the alignment.

### Superposition of individual domains

The BDV N protein consists of 370 amino acids, the N protein from VSV and RABV consists of 422 and 450 amino acids, respectively, and the FLUAV N (commonly known as NP) protein consists of 489 amino acids. There is no detectable homology at the amino acid sequence level among these N proteins. The structures of the VSV (PDB access code 2GIC) and the RABV (PDB accession code 2GTT) N proteins were superimposed with that of the BDV N protein (PDB accession code 1N93), respectively, by use of the FATCAT program with either rigid or flexible alignments [11]. Since the two N structures from the two rhabdoviruses are nearly identical, only the VSV N protein was used as the representative rhabdovirus N protein for subsequent analyses. The results of superposition of VSV N with that of BDV N are summarized in Table 2 and 3.

**Table 2:** Structural alignment of the C-terminal domains

RMSD (Å) ⇓ P-value Aligned residues	BDV N C-domain: (residues 230–346, 109 amino acids)	FLUAV N C-domain: (residues 213–271, 59 amino acids)
VSV N C-domain: (residues 224–341, 118 amino acids)	**3.44** **3.17e-5** **97**	**3.60** **2.49e-2** **46**
BDV N C-domain: (residues 230–346, 109 amino acids)		**4.23** **5.19e-3** **53**

⇓ For each paired comparison, three values were provided as listed in this box.

**Table 3:** Structural alignment of the N-terminal domains

RMSD (Å) ⇓ P-value Aligned residues	BDV N N-domain: (residues 50–229, 180 amino acids)	FLUAV N N-domain: (residues 21–202 [56–147]*, 73 amino acids)
VSV N N-domain: (residues 46–223, 178 amino acids)	**4.03** **3.90e-3** **133**	**3.61** **8.16e-2** **55**
BDV N N-domain: (residues 50–229, 180 amino acids)		**3.09** **1.33e-2** **45**

⇓ For each paired comparison, three values were provided as listed in this box.

*Note: When the N-terminal domain of the FLUAV N protein was superimposed with other N-terminal domains, only the core region (residues 56–147) was used in the superposition.

**Figure 2:**
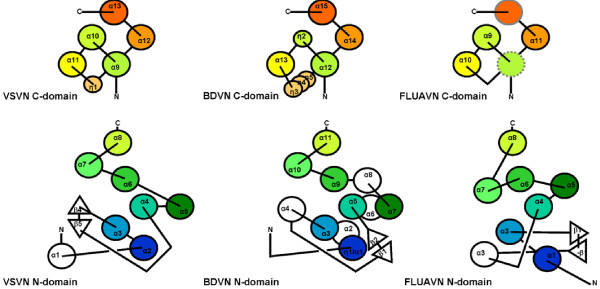
Topology drawings for the C-terminal domain (top panel) and the N-terminal domain of the N proteins. Large circles represent α-helices and triangles represent β-strands. Small circles represent 3_10 _helices. Color codes are from blue to red orange, corresponding to the sequence distance to the N-terminus similar as in Figure 4. Lines above the circles represent connections on top of the helices, whereas lines below the circles represent the connections at bottom of the helices. The secondary structure elements are labeled the same as in the reported crystal structures. The dotted gray colored circle in the FLUAN C-terminal domain implies a possible disordered α-helix and the gray circle implies a mismatch of a loop with an α-helix.

**Figure 3:**
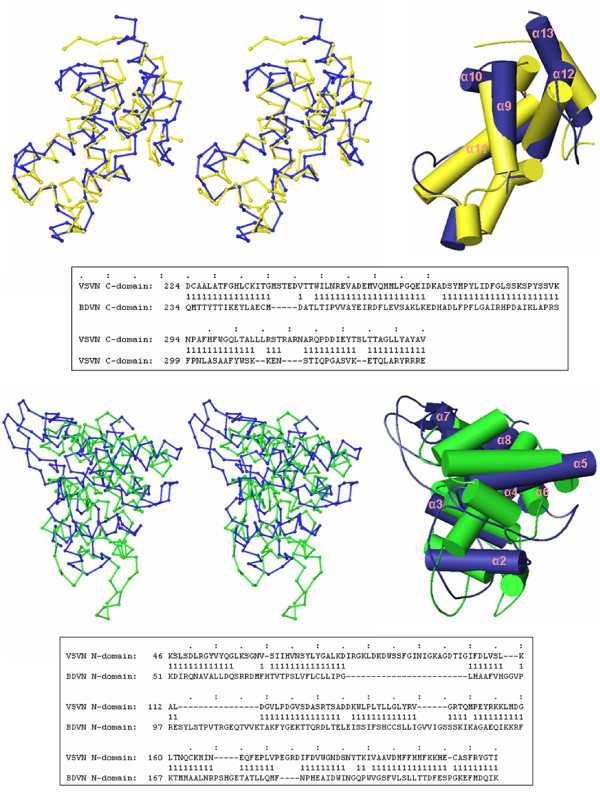
Comparisons of the VSV and BDV N structures. Stereo Cα drawings for the superposition of the C-terminal domain in the VSV N protein (blue) with that of the BDV N protein (yellow) (upper panel), and the N-terminal domain of the VSV N protein (blue) with that of the BDV N protein (green) (lower panel). Residue positions of the aligned structures are shown in the box below each structural comparison. '1' marks the aligned residues between the two structures. Cartoon drawings are also presented on the right with α-helices in VSV N labeled.

**Figure 4:**
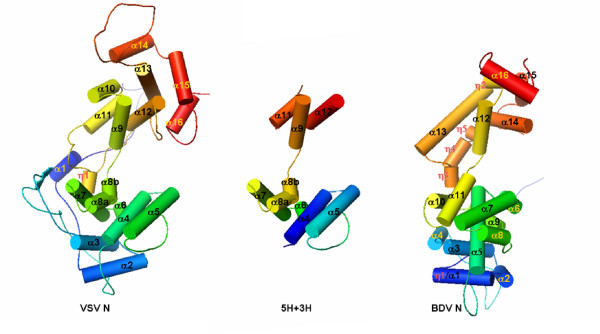
The structures of the VSV and the BDV N proteins compared to the (5H+3H) motif. The aligned helices are labeled by black lettering in each structure.

The topology of the protein fold is essentially the same between the VSV and BDV N structures each of which is composed of two domains (Figure 2). The central core of the N protein structure contains 7 aligned helices in the N-terminal domain and 5 aligned helices in the C-terminal domain. The N-terminal domain is directly linked by helix α8 to helix α9 in the C-terminal domain. The structure of the C-terminal domain is more conserved than that of the N-terminal domain (Figure 3, Table 2 and 3). The two domains may change their relative orientations in the RNA binding region if the N protein needs to encapsidate the RNA in a slightly different mode, such as binding a more or less number of nucleotides per N protein molecule. Nevertheless, in addition to the apparent topological similarity, the overall structure of the two N proteins has a very similar shape despite the difference in the orientation of the individual domains (Figure 4).

**Figure 5:**
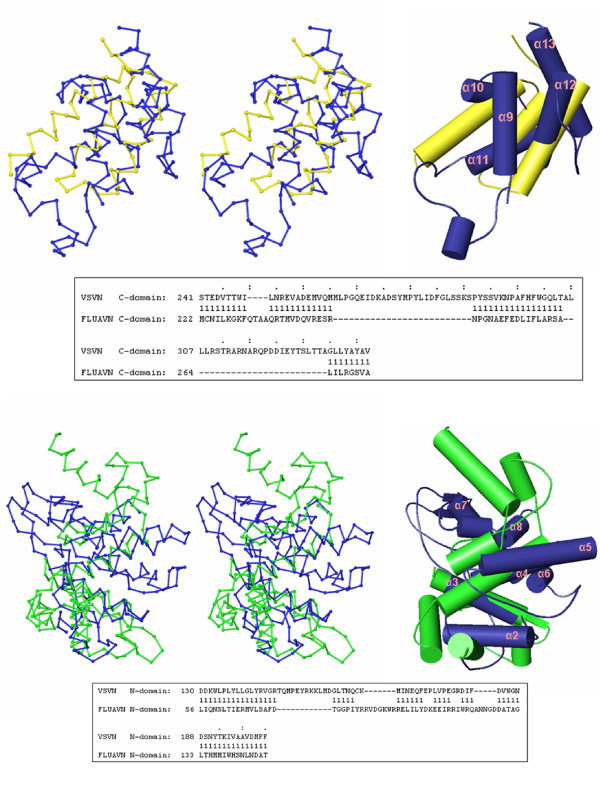
Comparisons of the VSV and FLUAV N structures. Stereo cartoon drawings for the superposition of the C-terminal domain of the FLUAV N protein (yellow) with that of the VSV N protein (blue), and the superposition of the N-terminal domain of the FLUAV N protein (dark green) with that of the VSV N protein (blue). The two domains in the FLUAV N protein were aligned with their counterparts in the VSV N proteins as separate domains. In the case of the N-terminal domain, only the core residues 56–147 were included in the calculation for structural alignment by FATCAT. Residue positions of the aligned structures are shown in the box below each structural comparison. '1' marks the aligned residues between the two structures. Cartoon drawings are also presented on the right with α-helices in VSV N labeled. Statistics for the domain alignments could be found in Table 1B and 1C.

A negatively charged surface groove was identified in the FLUAV N structure, but the RNA binding region was not clearly mapped in the previous report [6]. We found that the region comprised of residues 21–271 of the FLUAV N protein (PDB accession code 2IQH) is structurally similar to the RNA binding region of the VSV N protein, but the two domains within this region are positioned differently in the FLUAV N structure (see below). This shows that the two domains in the FLUAV N structure have a large change in their relative orientations compared to those in the VSV N structure. As a result, each domain in the two proteins could only be superimposed separately (Figure 5). The C-terminal domain again is structurally more conserved between VSV and FLUAV N proteins with secondary structure elements arranged by a similar topology (Figure 2, Table 2 and 3). The N-terminal domain of the FLUAV N structure could only be superimposed with that of the VSV or BDV N structure when the core residues 56–147 from the N-terminal domain were included.

Each N protein has other secondary structural elements that are not aligned (Figure 2). For instance, there is a long loop between the second and the third conserved α-helices (α3 and α4 in the VSV N protein) in the N-terminal domain, with a β-hairpin at the tip of the loop. This loop is also present in the N-terminal domain of the BDV N protein as shown by the flexible superposition of the two structures [11], but it is pointing to the opposite direction as a result of the insertion of an α-helix (α4 in the BDV N protein). The C-terminal end of the VSV N protein beyond the C-terminal domain includes three consecutive α-helices whereas that of the BDV N protein contains only one. There are 3_10 _helices between the third and fourth α-helices (α11 and α12 in the VSV N protein) in the C-terminal domain (three 3_10 _helices in the BDV N protein and one in the VSV N protein). Additional α-helices can be found in the BDV N protein (Figure 2). In the case of the FLUAV N protein, the region that is superimposable with the RNA binding region of the VSV N protein contains essentially the same number of secondary structural elements except for helix α13 of the VSV N protein. The rest of the FLUAV N protein (residues 272–489) has no homologous counterpart in either VSV or BDV N proteins. These residues constitute an additional domain near the C-terminal end of the FLUAV N protein.

### RNA binding cavity

**Figure 6:**
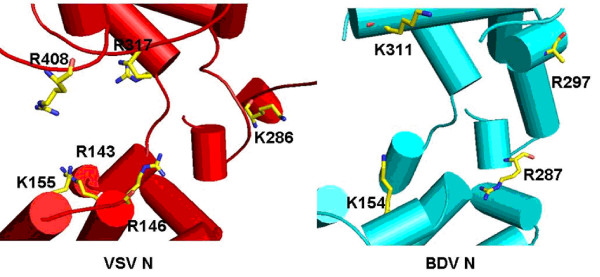
The RNA binding cavity of the VSV N protein (red) with highlighting the positively charged residues that interact with the RNA. For comparison, the similar region in the BDV N protein (cyan) was presented. Positively charged residues that could potentially interact with the RNA are also highlighted in the BDV N structure. The sidechain of Arg297 in the BDV N structure was disordered in the crystal structure (not shown in this figure).

Since structures of both BDV and FLUAV N proteins were determined in the absence of RNA, comparisons to the structure of the N-RNA complex of VSV could help in identifying the residues that interact with RNA. When the crystal structure of the BDV N protein was first published, the authors observed highly positively charged areas in the interior surface of the tetrametic BDV N protein. However, it appears that it is difficult to thread an RNA strand through the center of the tetramer. In fact, all the crystal structures of the N proteins reported so far were oligomers that are much smaller than the RNP, with or without RNA. The RNP has an extended structure with a large number of N protein molecules lining side-by-side on the genomic RNA, including the BDV RNP [13]. The interior of the RNA binding cavity in the rhabdovirus N protein is mostly hydrophobic, which accommodates the bases that point toward the N protein [4,5]. This hydrophobic region is also found in that of the BDV N protein, but comprising of nonhomologous residues. Six positively charged residues in the VSV N protein were identified to interact with the phosphate groups in the bound RNA, three in the N-terminal domain (Arg143, Arg146 and Lys155), and three in the C-terminal domain (Lys286, Arg317 and Arg408) (Figure 6). In the BDV N protein, four positively charged residues are located near those residues in the VSV N protein, one in the N-terminal domain (Lys154) and three in the C-terminal domain (Arg287, Arg297 and Lys311) (Figure 6B). If the C-terminal domains in the VSV and BDV N proteins are aligned as the anchor, the BDV N protein seems to have a cavity composed of 5 helices from the N-terminal domain and 3 helices from the C-terminal domain, with a similar size as that in the VSV N protein (Figure 5).

**Figure 7:**
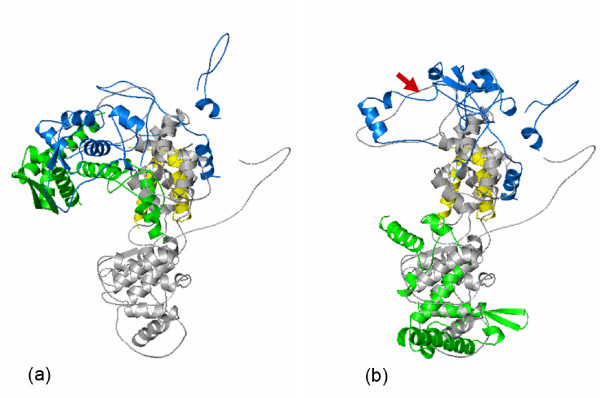
Cartoon drawings for the superposition of the FLUAV N protein with that of the VSV N protein (gray). (a) Superposition of the FLUAV N protein as in the reported crystal structure. Only the C-terminal domain of the putative RNA binding region of the FLUAV N protein was included in the calculation for the structural alignment by FATCAT [11]. The N-terminal domain of the putative RNA binding region in the FLUAV N protein was colored green, the C-terminal domain, yellow, and the addition domain at the C-terminal end of the FLUAV N protein, blue. (b) Superposition of a hypothetical structure of the FLUAV N protein with the structure of the VSV N protein. The orientation of the C-terminal domain is the same in (a) and (b). The N-terminal domain (green) was aligned with the N-terminal domain of the VSV N protein as in Figure 5. The additional domain at the C-terminal end (blue) was positioned by twisting the torsion angles of the peptide chain including residues 295–297 (indicated by the red arrow) to match the loop at the end of the VSV C-terminal domain.

The two domains of the FLUAV N structure corresponding to the RNA binding region of the VSV N protein have very different positions compared to the other structures (Figure 7A). For this reason, residues that may be similar to the RNA binding residues in the VSV N cavity could not be definitely identified. If the C-terminal domain of the FLUAV N protein is aligned with that of the VSV N protein, the N-terminal domain and the additional domain at the C-terminal end of the FLUAV N protein would close the cavity that is present in the rhabdovirus N protein (Figure 7A). The structural comparisons discussed above have shown that the two domains are separately conserved structural domains and may assume various orientations relative to each other. It is not unacceptable that the orientations of the two domains in the FLUAV N protein in an RNA-free conformation may be changed in an RNA-bound conformation. To explore that possibility, an open conformation was simulated by aligning each domain individually, i.e. the N-terminal domain and the C-terminal domain of the FLUAV N protein were aligned with those of the VSV N protein as in Figure 5. Next, the additional domain at the C-terminal end is manually positioned to match the extreme C-terminal end of the VSV N protein. This maneuver requires only rotations (twists) of two clearly defined structural domains in the FLUAV N protein. The final simulated open conformation of the FLUAV N protein (Figure 7B) is essentially derived from the N conformation that is observed in the VSV N-RNA complex.

### Oligomerization

The N protein polymerizes on the genomic RNA during replication. Neighboring N molecules form an extended network of interactions along the entire length of the RNA genome. In the VSV N protein, there is a 1954 A^2 ^buried area side-by-side between two monomers while the buried area is 2680 A^2 ^in the BDV N protein. The larger buried area in the BDV N proteins could be the result of the tetrameric oligomerization, which has a 90° angle between two neighboring molecules compared to an angle of 144° or 147° for the rhabdovirus N proteins that were crystallized as a 10 mer and 11 mer, respectively, with RNA bound. The BDV N protein molecules would have to associate through more extended side-by-side interactions in the RNP, which should have similar contact areas between the neighboring

N molecules as observed in the rhabdovirus N-RNA complexes. The extended C-terminal and N-terminal arms in both structures reach over the neighboring molecules to add further intermolecular interactions. The oligomerization arrangement of the reported FLUAV N structure [6] is so different that it is impossible to make a meaningful comparison of the reported FLUAV N oligomer with that of the rhabdovirus or BDV N proteins. Comparisons of how the interactions between the FLUAV N proteins contribute to encapsidation of the RNA genome would become more apparent if a structure of the FLUAV N-RNA complex becomes available.

## Testing the hypothesis

Comparisons of the N protein structures from three virus families showed that the RNA binding region in each N protein has a similar structure containing two domains. The overall structure of the rhabdovirus N protein can be superimposed with that of the BDV N protein, whereas the FLUAV N protein could only be superimposed with the other N proteins as separate N-terminal and C-terminal domains. However, it appears that the fold of the individual domains are conserved in the N proteins to a degree similar to that of the β-barrel fold in the capsid proteins of spherical viruses. There are five helices in the N-terminal domain and three helices in the C-terminal domain that are common among the N structures of the three virus families. This motif, which we have named the (5H+3H) motif, may be a common motif responsible for encapsidating RNA by the N protein of negative strand RNA viruses (Figure 2). The helices α8 and α9 named as in the VSV N protein are at the center of the motif and connect the two domains in the motif. However, the spatial geometry of the helices in the (5H+3H) motif is variable when the structures were compared. One possible explanation for this observation is that the structures of the BDV and FLUAV N proteins were determined without RNA bound [3,6] whereas those of the VSV and RABV N proteins were determined with a random RNA molecule bound in the RNA binding cavity [4,5]. The orientation of the helices in the BDV or FLUAV N protein might change when the N protein binds RNA. This question could be answered when the structure of the BDV or FLUAV N protein is determined in the presence of bound RNA. An alternative explanation could be that there are intrinsic differences in the three dimensional structure of the N proteins, a likely result of evolution despite commonality of the structure and function of the N proteins among negative strand RNA viruses.

## Implication of the hypothesis

The structural alignments of the N proteins from three negative strand RNA virus families have significant predictive values in recognizing the RNA binding site and the side-by-side interactions of the BDV N protein, which was not revealed when the BDV N structure was determined alone in the absence of bound RNA. The chemical properties of the homologous cavity in the BDV N protein and the pattern of intermolecular interactions are consistent with its functions to assemble the viral RNP. It also suggested a possible conformation of the FLUAV N protein which may be more suitable for RNA binding than the conformation observed in the recent crystal structure.

## Competing interests

The author(s) declare that they have no competing interests.

## Authors' contributions

ML, TJG, XZ, JT, SQ generated data used in the analysis, proposed and discussed the hypothesis. ML and TJG wrote the manuscript. All authors read and approved the final manuscript.
